# TiCrN-TiAlN-TiAlSiN-TiAlSiCN multi-layers utilized to increase tillage tools useful lifetime

**DOI:** 10.1038/s41598-019-55677-8

**Published:** 2019-12-13

**Authors:** Shahab Sharifi Malvajerdi, Ahmad Sharifi Malvajerdi, Majid Ghanaatshoar, Morteza Habibi, Hassan Jahdi

**Affiliations:** 1 Laser and Plasma Research Institute, Shahid Beheshti University, G. C. Evin, Tehran, 1983963113 Iran; 2 Agricultural Engineering Research Institute, Agricultural Research, Education and Extension Organization (AREEO), Karaj, Iran; 3 Energy Engineering and Physics Department, Amirkabir University of Technology, Tehran, 158754413 Iran; 4 Sevin Plasma Surface Engineering Co., Isfahan Science and Technology town, Isfahan, Iran

**Keywords:** Mechanical properties, Surfaces, interfaces and thin films

## Abstract

For the first time, a hard wear-resistant multi-layer of TiCrN-TiAlN-TiAlSiN-TiAlSiCN was deposited on carbon steel CK45-based tillage tools to increase their useful lifetime. The layers were deposited by using an arc-PVD method without post-annealing procedures. XRD and EDX data indicated that TiCrN, TiAlN, TiAlSiN, and TiAlSiCN formed individually and as a multi-layer of high-quality crystalline layers with mostly cubic structures. The studies on the multi-layers coating morphology, roughness and hardness gave reasonable results as a roughness of 35 nm and a hardness of 32.2 GPa. The coated sweep duck blade tillage tools were tested on the field along with a soil bin to obtain their wear behavior at different traveling distances. The draft force of all blades showed promising results. As the coated layers were worn off, their draft force increased. In comparison with single-layer coatings, the multi-layer structure demonstrated an increase in the useful lifetime of the blades.

## Introduction

Titanium-based composites have been of interest of researchers due to their suitable low friction coefficient, high thermal stability, and resistance to oxidation and wear^1,2^. These characteristics have made these materials good candidates for hard coatings to increase the lifetime of instruments utilized for harsh environments^3,4^. Most recently, single layer composite coatings such as TiAlN, TiAlSiN, and TiAlSiCN have been investigated for their well-improved mechanical properties compared to TiN coatings^5–8^. Multi-layer coatings have also gained researchers’ interest due to their advantages to single layer coatings^9–12^. Durmaz and Yildiz exhibited high-quality TiAlN coatings which had high wear resistance^13^. Even more, research on TiAlSiN coatings has proved suitability of this composite for anti-wear coating applications^14,15^. A research conducted by Shizhi *et al*. revealed that by adding Si (10–15%) to TiN will form TiSiN thin films leading to layers with higher hardness than TiN^16^. Other studies also proved that the presence of Si in TiN has a significant role in achieving very high hardness compound with better wear resistance^17^. These line-of-ceramic composites have proven to be well applied to high-temperature resistance turbine and aircraft engine blades^18,19^, high resistance to wear for dry cutting tools^20–23^, and other coating applications for molds and dies^24^, plowshares^25^ and tillage tools^26^. The utilized deposition method plays the key important role in achieving high-quality crystalline layers. TiN-based materials have been deposited by many different techniques over the past years. To name a few, Magnetron sputtering^27–32^, CVD^33,34^, PECVD^35^, multi-purpose plasma immersion ion implantation^8,36,37^, Plasma focus^38^, PVD^39,40^ and arc-PVD^41–44^.

The arc-PVD method is able to form high-quality thin films at low temperatures. The PVD method has been employed to grow many multi-refractory metal compositions for wear, thermal and corrosion resistance applications. For instance, TiAlSiN thin films were deposited by Yu, *et al*.^45^ who studied the hardness of the layers by changing the Si content ratio. This material was also deposited by Li, *et al*.^35^. Sui, *et al*.^7^ research on TiAlN-TiAlSiN double layers also revealed a high level of hardness of the coated tools for cutting applications. Along with research conducted on TiAlSiN, TiAlSi^46^, TiAlN^30,47–50^ and TiN-TiAlN^44^ multi-layers have also been deposited by arc-PVD method, which have shown promising mechanical properties. For many years, researchers investigated simple single layer coatings to increase wear and oxidation resistance of cutting tools. Nowadays, studies on hardness, wear, and corrosion resistance are being conducted on more complex and nanocomposite multi-layers to obtain a better performance. Recent investigations on TiAlSiCN multi-layers^51–53^ have shown their potential to be applied upon cutting tools to resist high temperature, corrosion and wear, and eventually to increase the useful lifetime of the tools.

One of the hardening applications of thin film deposition is in agriculture, where wear resistant thin films are deposited on tillage tools to obtain better performance and longer lifetime. Tillage tools are the core soil engaging implements which confront high abrasive wear. The processes by which tillage tools wear evidently include impact, abrasion and chemical actions^54^. Sweep blades are the most exploited soil preparation tools which are used on the combined tillage implements. Tillage tools should be strong enough to resist impact and wear and to work in hard soil conditions^54^. The case of single ceramic composite coated layers has been previously investigated to reduce wear^1,55^. In our previous research on TiN single layer coating, we were able to obtain resealable results in increasing the lifetime of sweep duck blades^1^. Some of other hardening techniques applied to tillage tools are alumina ceramic coatings^56^, hard facing^57^, plasma restoration and hardening^58^, carbo-vibro-arc hardening method^59^, and hot stamping processes^60^.

In this research, we aim to deposit TiCrN-TiAlN-TiAlSiN-TiAlSiCN multi-layer for the first time, and eventually use them to increase the useful life of sweep duck blade tillage tools. With the procedure of stacking four different layers with similar crystal structures, we aim to introduce a multi-layer with higher wear resistance, hardness and longer lifetime than single layer TiN^1^. The sweep duck blades (carbon steel CK45) will be coated with TiCrN, TiAlN, TiAlSiN, TiAlSiCN layers using an arc-PVD system with no post-annealing process, before or after the deposition. The first layer, TiCrN, is used as a base layer for other layers with higher hardness to be able to be deposited on top of the carbon steel CK45. The multi-layer has been designed in a way that the hardness of the layers increases from the bottom to the top. The experiments on the coated blades will be carried out at the laboratory and real field conditions to obtain more detailed results for both researchers and manufacturers. In order to examine the performance of the coating, the blades are sent to loamy condition soil. They are tested at four different traveling distances by a tractor, and subsequently, they are tested in a soil bin laboratory condition to measure their draft force. Then, samples from the blades are taken apart to be studied on. Scanning electron microscopy (SEM) and atomic force microscopy (AFM) are employed to study the surface morphology and topology of the blades before and after the field experiment. Energy dispersive X-ray spectroscopy (EDX), and X-ray diffraction (XRD) are exploited to indicate the elements and the crystallinity of the thin films. Additionally, the surface hardness and its roughness are studied by a Vickers hardness monitor and a profilometer. We employ a penetrologger to measure soil mechanical resistance. Soil moisture content is obtained using an oven along with soil texture and chemical properties which are investigated by EC meter, pH meter, Anion-Cation spectrophotometer, and a hydrometer.

## Results

### XRD

**Figure 1:**
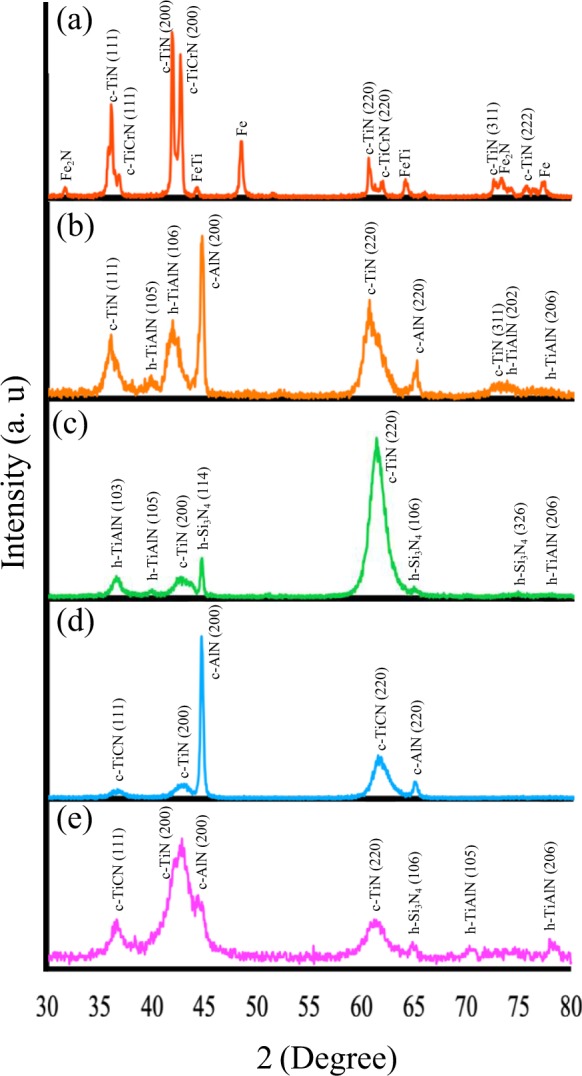
XRD data of (**a**) TiCrN, (**b**) TiAlN, (**c**) TiAlSiN, (**d**) TiAlSiCN single layers, and (**e**) the multi-layer.

XRD patterns of all individual layers and the whole multi-layer are shown in Fig. 1. The XRD data indicated that mostly cubic structures of the materials were formed along with only a few hexagonal structures. In the first layer (TiCrN), two main crystal structures formed; c-TiN (111), (200), (220), (311), (222), and c-TiCrN (111), (200), and (220). According to Fig. 1a there are also other peaks which belong to Fe, Fe_2_N, and FeTi through Ti and N bonding to the Fe atoms of the substrate. The second layer, TiAlN, had three crystal structures; c-TiN with (111), (220), and (311) peaks, h-TiAlN with (105), (106), (202), and (206) peaks, and c-AlN with (200) and (220) peaks (Fig. 1b). Due to the XRD pattern in Fig. 1c, h-TiAlN {(103), (105), and (206)}, c-TiN {(200), and (220)}, and h-Si_3_N_4_ {(114), (106), and (326)} are crystal structures that formed in the third layer, TiAlSiN. XRD of the final layer TiAlSiCN indicated three structures (see Fig. 1d). They are c-TiCN (111) and (220) planes, c-TiN with (200) plane, and c-AlN with (200) and (220) planes. Figure 1e shows the XRD of the multi-layer by which many peaks from all 4 layers were detected. They are c-TiCN with (111), c-TiN (200), and (220), c-AlN (200), h-Si_3_N_4_ (106), and h-TiAlN (105), and (206). The XRD data was processed by an Xpert High Score Plus software. The corresponding data sheet reference codes are 01–087–0631 for c-TiN, 01-070-2981 for c-TiCrN, 01-089-4185 for Fe, 03-065-7743 for FeTi, 00-006-0656 for Fe_2_N, 01-080-2286 for h-TiAlN, 00-046-1200 for c-AlN, 01-079-2011 for h-Si_3_N_4_, and 01-076-2484 for c-TiCN.

All crystallite sizes were calculated from full width of half maximum (FWHM) of the XRD peaks by using the Debye-Scherrer equation (*τ* = *Kλ*/*βcosθ*)^61,62^ (*τ* is the size of the crystallite, K is Scherrer constant 0.94, λ is the X-ray wavelength which in this case is 1.54Å produced by a copper pole, *β* is Δ(2*θ*) (FWHM) of the XRD peaks, and *θ* represents the angles where the peaks are positioned^63^). The crystallite sizes of the crystal structures formed in the bottom layer were calculated to be 30 nm for c-TiN and 30 nm for c-TiCrN, in TiAlN layer; 12 nm for c-TiN, 6.5 nm for h-TiAlN, and 22 nm for c-AlN, in the third layer, TiAlSiN; 9.8 nm for c-TiN, 11.1 nm for h-TiAlN, and 22.73 nm for h-Si_3_N_4_, and in the fourth layer (TiAlSiCN) crystallite sizes where 6.47 nm for c-TiN, 6.14 nm for c-TiCN, and 16.7 nm for c-AlN. The crystallite sizes were also calculated from the XRD data of the multi-layer that were 5.66 nm for c-TiN, 11.11 nm for c-TiCN, 15.2 nm for c-AlN, 16.65 nm for h-Si_3_N_4_, and 12.91 nm for h-TiAlN.

### EDX and Line scan

**Figure 2:**
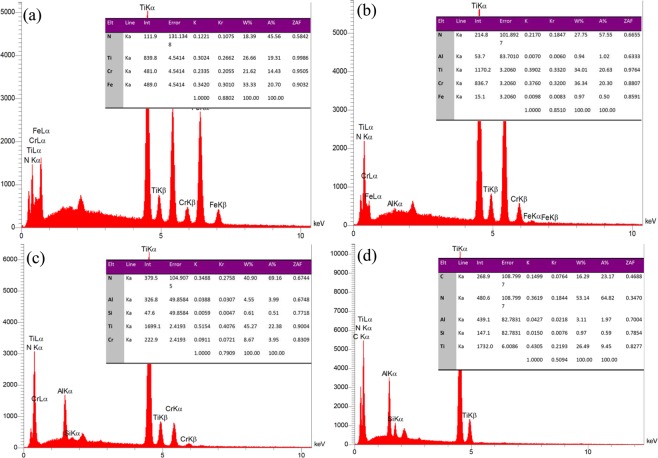
EDX spectra of (**a**) TiCrN, (**b**) TiAlN, (**c**) TiAlSiN and (**d**) TiAlSiCN layers in the multi-layer.

**Figure 3:**
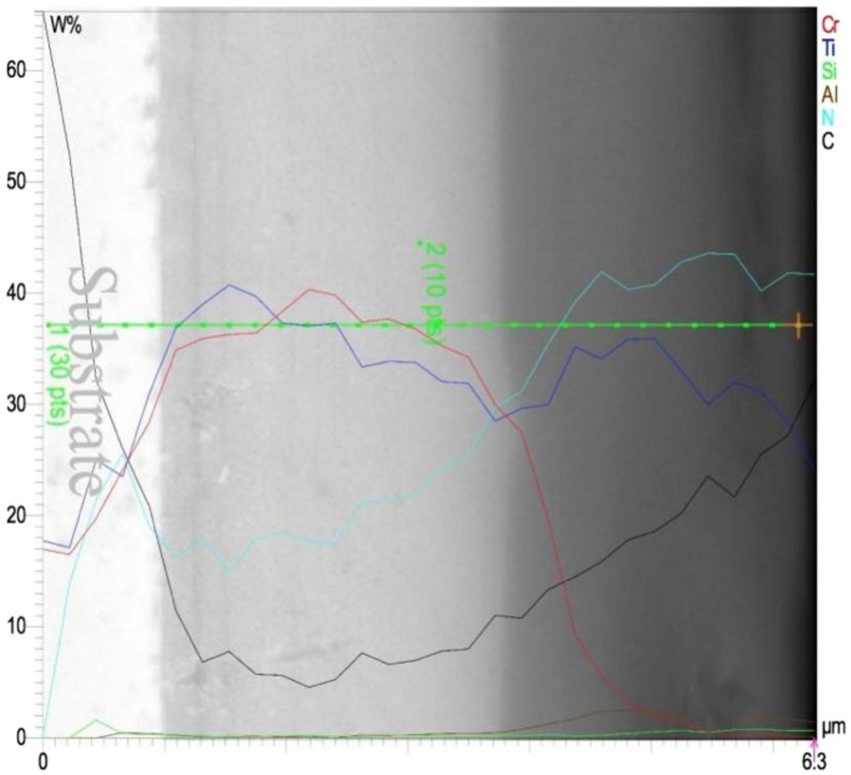
EDX line scan of the multi-layer.

The elements of each individual layer and their quantification were obtained by EDX cross-sectional spectrum (Fig. 2). This set of data backs up the XRD patterns to conclude that the TiCrN, TiAlN, TiAlSiN and TiAlSiCN layers have been perfectly formed. Figure 2a shows the element count of the first layer, TiCrN, where the atomic weight of Ti and Cr elements match almost at 50:50 ratio as the deposition procedure took place with the same rate of deposition for both materials. In Fig. 2b, EDX spectrum reveals Ti, Cr, Al, and N atoms with 1% atomic Al amount. The Cr detected in this layer has been originated from the first layer, shown in cross-sectional image. Moreover, the Fe in the first two layers is originated from the substrate. Therefore, the ratio of Al to Ti is enough to form TiAlN. The third layer EDX quantification in Fig. 2c indicates Ti and N 50:50 ratio along with 4% Al and 0.5% Si. Finally, the elements of TiAlSiCN layer were observed to be 10% Ti, 23% C, 2% Al, and 0.6% Si (Fig. 2d). Along with the single layer EDX a Line scan of all layers was obtained to detect all elements and their distribution throughout all the layers. The data in Fig. 3 indicates that the deposition of all the respective layers at 400 °C was carried out well enough to form hard ceramic layers.

### SEM

**Figure 4:**
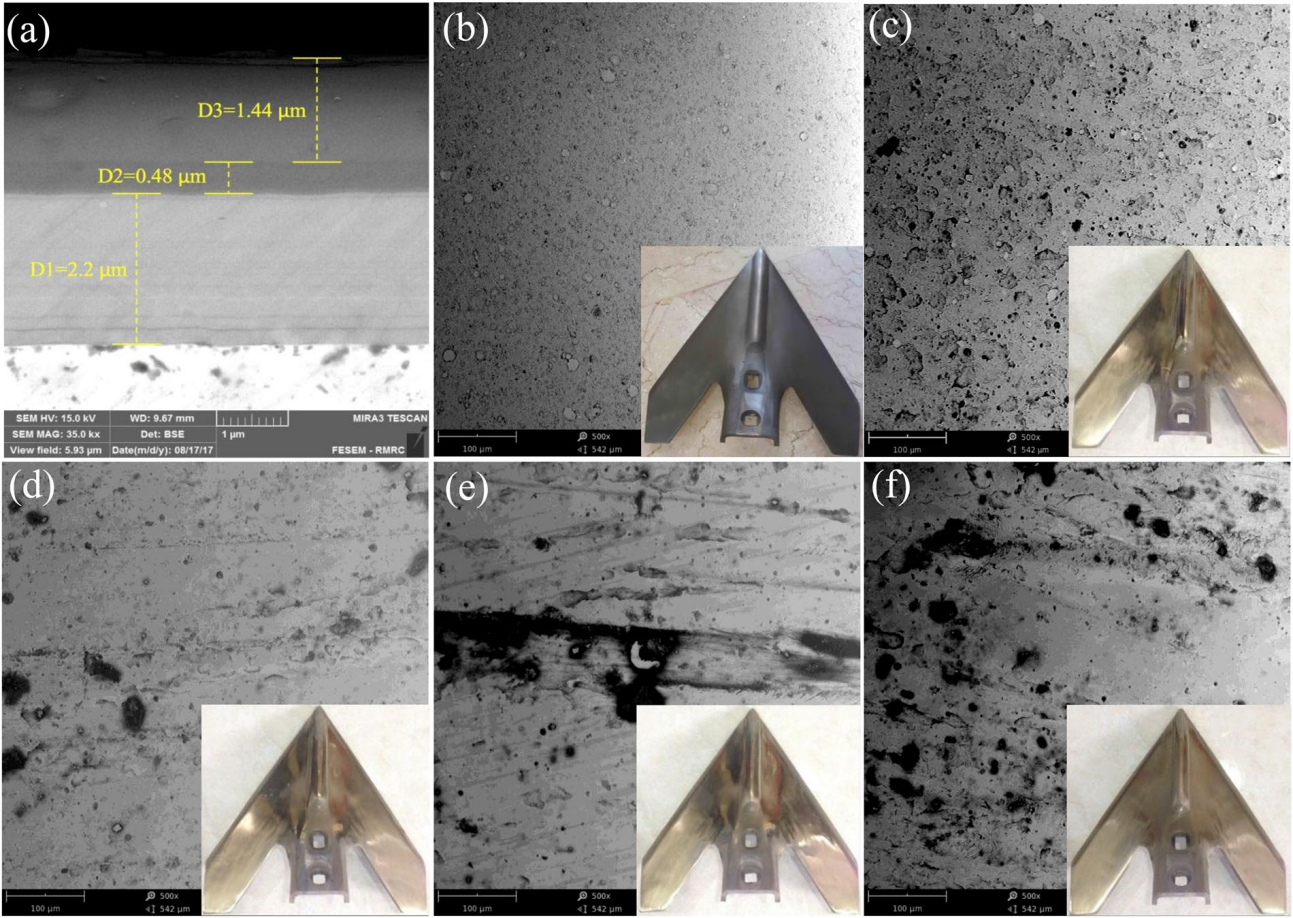
SEM images of the samples before and after the experiment. (**a**) SEM cross-sectional image of the layers deposited on a polished sample. (**b**) The surface topography of the coated sample before the experiment. Other figures represent the SEM images of the samples that traveled (**c**) 2 km, (**d**) 4 km, (**e**) 6 km, and (**f**) 8 km.

Figure 4 shows the blades along with SEM images which illustrate surface morphology of the deposited layers before and after the experiment. Image of the TiCrN-TiAlN-TiAlSiN-TiAlSiCN multi-layer surface of the coated blade is shown in bottom corner of Fig. 4b. The cross-section SEM image (Fig. 4a) shows the thickness of each individual layer. TiCrN layer is approximately 2.2 μm thick, and TiAlN thickness is ~0.48 μm. The two last layers TiAlSiN and TiAlSiCN at the top, have a whole thickness of around 1.44 μm. The reason that the two last layers cannot be distinguished from each other is the resolution of the SEM instrument. However, the line scan analysis in Fig. 3 confirms the formation of all the layers. After the experiment took place, the surface morphology of all the samples were investigated. The optical image of the blades in the bottom corners of Fig. 4b–f reveals that only the top layer which is TiAlSiCN with dark gray color has been worn off. The last sample, which traveled 8 km, clearly shows that the deposited layers perfectly resist to wear under the hard-rocky soil conditions.

### Hardness

The hardness measurement was carried out by a Vickers hardness monitor. The hardness of the 4.2 μm-thick TiCrN-TiAlN-TiAlSiN-TiAlSiCN multi-layer was measured to be 32.2 GPa with 15 g applied weight. The hardness of each individual layer alone was 23.7 GPa for TiCrN, 25.8 GPa for TiAlN, 34.6 GPa for TiAlSiN, and 36.4 GPa for TiAlSiCN.

### Roughness

**Figure 5:**
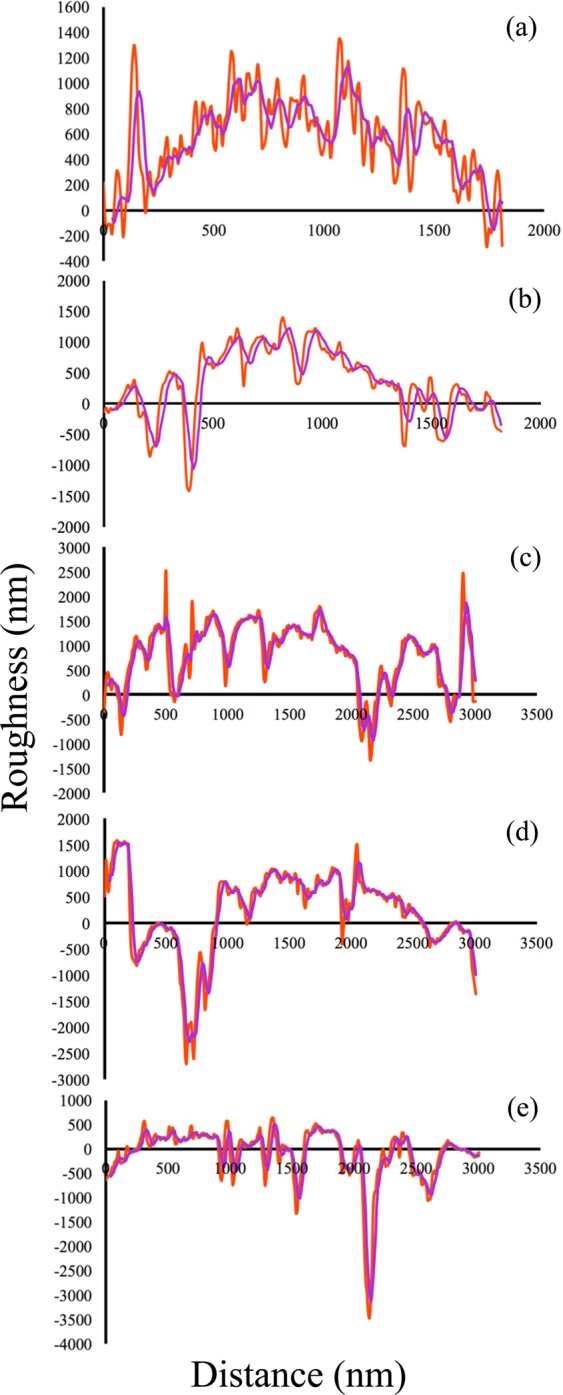
Surface profile of the coated samples before and after the experiment. The orange line stands for the profile and the purple line displays the profile moving average (**a**) before experiment and for (**b**) 2 km, (**c**) 4 km, (**d**) 6 km and (**e**) 8 km traveled blades.

3D AFM and 2D profilometer surface roughness investigations were conducted, both before and after the blades were examined in the field. Figure 5 shows the moving average (purple lines) of the profiles along with their roughness arithmetical mean deviation (R_a_), roughness root mean squared (R_q_), maximum valley depth (R_v_), maximum peak height (R_p_) and maximum height of the profile (R_t_), which determines the approximate thickness of the layer (Table 1)^64^. These parameters are defined as follows:

1 $${R}_{a}=\frac{1}{n}\mathop{\sum }\limits_{i=1}^{n}|{y}_{i}|$$

2 $${R}_{q}=\sqrt{\frac{1}{n}\mathop{\sum }\limits_{i=1}^{n}{y}_{i}^{2}}$$

3 $${R}_{v}=\mathop{\min }\limits_{i}{y}_{i}$$

4 $${R}_{p}=\mathop{\max }\limits_{i}{y}_{i}$$

5 $${R}_{t}={R}_{p}+{R}_{v}$$

**Table 1:** Profilometer measurements of the blades before and after the field experiment.

Distance (km)	0	2	4	6	8
R_a_ (nm)	211	182	357	576	231
R_q_ (nm)	4167	3866	8637	7863	3473
R_v_ (nm)	−154	−1052	−933	−1537	−505
R_p_ (nm)	1125	1227	1858	1537	505
R_t_ (nm)	1279	2280	2792	3822	3638
STDEV	345	551	654	779	573

### Draft force and duncan’s test

**Figure 6:**
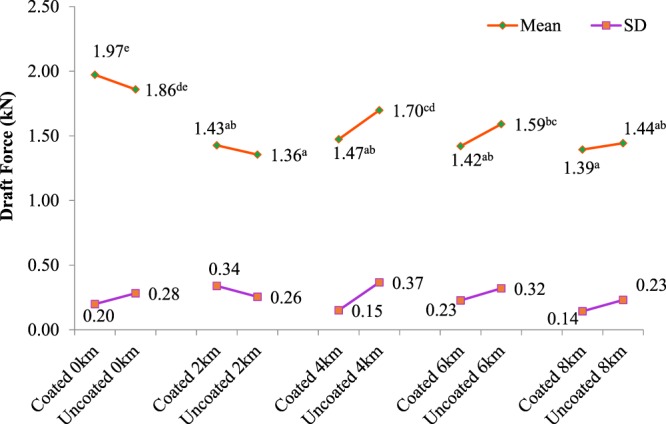
The draft force of the coated and uncoated blades at travelled distances of 0, 2, 4, 6 and 8 km.

The mean values of draft force of the blades at various distances obtained from the experiment conducted in a soil bin are presented in Fig. 6. These values are corresponded to coated and uncoated blades. The standard deviations of the means are also given to show the mean variations of the data of draft forces. Although the draft forces of coated blades at 0 km (before file experiments) and 2 km distances is more than that of uncoated ones but the coated blades caused less draft force than uncoated ones with the values of 1.47, 1.42 and 1.39 kN consumed by tractor than uncoated blades at the traveled distances of 4, 6 and 8 km in an agricultural field, respectively. As the travel distance increases the draft force decreases for multilayers TiCrN-TiAlN-TiAlSiN-TiAlSiCN coated blades. The mean values of draft force should have been statistically analyzed. Therefore, data were analyzed by Duncan’s multiple ranges test and the results are given in Fig. 6. There is no significant difference between the mean values with same letters at 95% level of confidence (Fig. 6). The results of the variance analysis (ANOVA)^65,66^ is also shown in Table 2. The effect of distance on draft force was significant. Specifically, the interaction effect of traveled distance and coating was significant (p < 0.05).

**Table 2:** ANOVA table of coated and uncoated blades data.

Source	Sum of square	df	Mean square	F	Sig
Model	74.551^a^	10	7.455	680.414	0.000
Travelled distance	1.078	4	0.270	24.600	0.000
Coating	0.017	1	0.017	1.577	0.224
Travelled distance × Coating	0.135	4	0.034	3.078	0.040
Error	0.219	20	0.011		
Total	74.770	30			

^a^R Squared = 0.997 (Adjusted R Squared = 0.996).

## Discussion

The XRD (Fig. 1) data demonstrated that the arc-PVD method without the need of post-annealing was successful in forming crystallites of c-TiN, c-TiCrN, c-TiCN, and c-AlN along with two hexagonal structures of h-TiAlN and h-Si_3_N_4_. c-TiN (200) peak was detected in all layers but its intensity decreased as c-AlN and h-Si_3_N_4_ formed in the other layers. This can be the result of the formation of polycrystalline h-Si_3_N_4_ in the third layer (Fig. 1c) and amorphous Si_3_N_4_ in the fourth layer (Fig. 1d) This is similar to results obtained by chen *et al*.^8^. In their TiAlSiN layer c-TiN peak intensity was reduced due to the presence of Si and the formation of amorphous Si_3_N_4_. The most intense peak belonging to TiN (200), showed in Fig. 1 was seen in Wei Li’s *et al*. and Peng’s *et al*. research^29,67^. As well as the mentioned structures, h-TiAlN formed without post-annealing (Fig. 1b). In the work conducted by Endrino *et al*.^40^, in which TiAlN thin films were deposited by a cathodic arc implanting method, both hexagonal and cubic phases of AlN and cubic TiN crystallites were obtained upon post-annealing and no exact detection of TiAlN structure became possible. This approves of the fine quality of the layers obtained at low temperature by the arc-PVD method and illustrates that the arc-PVD method has advantages to other methods that need post-annealing at high temperatures. The XRD data observed in Xudong Sui, *et al*. attempt^7^ at depositing TiAlN-TiAlSiN by reactive magnetron sputtering method at 400 °C had TiAlN (111) peaks which were not similar to the peaks observed in Fig. 1b,c.

It is notable that in comparison with single layers, the crystallite size for all structures has been reduced in the multi-layer and only c-TiCN and h-TiAlN crystallite sizes have been increased. As the results show, the crystallite sizes have also reduced by the presence of Si and Al atoms in the TiAlN, TiAlSiN, and TiAlSiCN thin films. The same issue was reported in previous study where the reason for crystallite size reduction was discussed to be due to the presence of amorphous SiN_x_ in TiAlSiN thin films^10^. It is notable that in recent attempts on the growth of TiN-based layers^7,9,14^ XRD patterns were different with much less intense peaks than the data in Fig. 1 which was deposited by an arc-PVD. Furthermore, the XRD spectra of our samples (Fig. 1d,e) have no similarity to that of the TiAlSiCN layer deposited which was by a DC magnetron sputtering and annealed at high temperature in Kuptsov’s *et al*. study^27^.

The SEM image of the surface of the coated blade before the experiment exhibited a uniform deposited layer (Fig. 4b). As the resistance to wear of the samples was tested (Fig. 4c) at 2 km, inhomogeneous layers that had carbon began to wear off. Going forward toward the beneath layers we observed that the homogeneous hard layers of TiAlSiN and TiAlN withstood the wear test with only a few worn off sections at 4 to 8 km (Fig. 4d–f). It is of importance to note that only small sections on the tips and edges of the blades wore off and the main body revealed high resistance to wear as shown in the optical images of the all blades in Fig. 4. Comparison of SEM images in Fig. 4 to samples that were annealed after deposition in other studies such as, the work performed by Golizadeh *et al*.^6^ is notable. Kuptsov *et al*. in a research on TiAlSiCN layers which were deposited by a DC magnetron sputtering and annealed at high temperatures^52^, could not obtain uniform stacked layers like the ones grown by using the arc-PVD method in our study (Fig. 4). Moreover, the images were taken after the experiment had shown much better results than those of the research conducted by Nalbant^26^ and also our previous research^1^, where a single TiN layer was used to incearse the lifetime of sweep duck blades. The TiCrN-TiAlN-TiAlSiN-TiAlSiCN multi-layers resulted in a much better and improved performance than the TiN layers which were applied to tillage blades^1,26^. While the TiN layers, which were deposited by a PVD method, totally wore off at a distance of around 4 km in Noblbant’s *et al*. studies^26^, in our experiment the arc-PVD layers did not wear off even after 8 km traveling distance on the farm. It is obvious that TiN-based arc-PVD coatings in this research have shown advantages to the layers, deposited by other created techniques.

R_a_ of the multi-layer deposited by the arc-PVD was obtained 35 nm by the AFM while other studies reported it about 32 nm^51^ and 25 nm^52^ for TiAlSiCN single layer coatings. Thickness reduction and an increase in roughness of all samples were observed after the field experiment. The SEM cross-section image calculated the overall thickness to be 4.2 μm (Fig. 4a). Thus, the thickness reduction (worn layer) of each sample after the experiment can be calculated by subtracting the overall thickness from R_t_. Table 1 shows that R_t_ of the sample traveled 2 km was about 2280 nm. This indicates that the worn layer was approximately 1720 nm. It can be concluded that TiAlSiCN layer nearly wore off after 2 km travelling. For the samples that traveled 4, 6, and 8 km, the worn layer was measured to be ~1208 nm, ~178 nm, and ~362 nm (Table 1), concluding that the resistance to wear of the samples were higher as the traveled distance was increased. Practically, the layers with high hardness showed high resistance to wear on the field.

The hardness of our arc-PVD-deposited multi-layer sample with no post-annealing procedure at 32.2 GPa was comparable to the hardness of the layers created by Golizadeh *et al*.^6^. They obtained their hard TiAlSiCN layers at ~22 GPa by post-annealing at high temperatures. In another report made by Bondarev *et al*.^53^, they could achieve TiAlSiCN single layers with hardness of about 41 to 49 GPa by vacuum post-annealing at 1000 °C.

Numerous researches regarding coating tillage tools have been conducted by different scientists. Some of which have used various coating material to investigate their impact on wear rate, useful lifetime and draft force, i.e. TiN, Nickle, chromium, aluminum, and plastic compounds for coating tillage tools. We discuss the results of previous works relevant to the effect of coatings on draft force of tillage tools confirming the results of the present study in this section. Nickel-based alloy powder was used for coating sweep blades to investigate its impact on draft force in a soil bin by Kushwaha *et al*. Draft force of the blades reduced by 15% in comparison with the uncoated blades^54^ and^68^. Other research works have shown decrement on draft force acting on the coated tillage tools. For example, new hard facing materials (carbon nanotube-hard chromium composite coated on tillage tools caused 43.29% reduction on draft force of tools^69^. In comparison with the previous works as mentioned above, our work also demonstrated in agreement with the other studies reduction of averagely 9% in draft force of the coated blades compared to uncoated ones at 4, 6 and 8 km distances. In this case, TiCrN-TiAlN-TiAlSiN-TiAlSiCN multi-layers have impacted the blades draft force significantly as they have reduced draft force after 4 km along with the wear resistance and useful lifetime increment.

## Experimental Methods and Conditions

A cathodic arc evaporation PVD (JHDI/Quad/01 arc-PVD Sevin Plasma Surface Engineering Co.) was used to deposit TiCrN-TiAlN-TiAlSiN-TiAlSiCN multi-layers on carbon steel CK45 substrates (sweep duck blades). Four sweep duck blades were sandblasted with Al_2_O_3_ to sweep away the paint on manufactured painted sweep duck blades. Subsequently, the blades were polished. The samples were heated up to 400 °C in a vacuum pressure of 10^−5^ mbar in the arc-PVD chamber. After the substrates reached the intended temperature, 99.999% nitrogen gas (N_2_) was inserted into the chamber that increased its pressure to 10^−3^ mbar. The coating targets, 99.9% Ti and 99.9% Cr were biased up to 40 V and the deposition was undergone for 80 min to form TiCrN thin film. Following the TiCrN layer formation of about 2.2 μm, the chamber was cleaned with argon (Ar) gas. The second phase of the deposition began with the same working gas (N_2_), vacuum pressure, biasing voltage, and deposition time, but in this stage for the formation of the second layer a 99.9% TiAl (Ti 50%, Al 50%) target was used. For the deposition of the third layer, TiAlSiN after the cleaning procedure by Ar gas, the samples were coated using 99.9% TiAl (Ti 50%, Al 50%) and AlSi (Al 80%, Si 20%) targets with the same conditions mentioned in the first 2 stages. Finally, for the fourth layer, TiAlSiCN, the same targets and conditions as the third stage were utilized but in order to add carbon to TiAlSiN, a mixture of methane (CH_4_) and N_2_ (CH_4_ 50%, N_2_ 50%) was inserted as working gas.

A combined tillage tool equipped with the sweep duck blades was used for the field experiments. Experiments were conducted in an agricultural field located at 51° 6′ longitude and 35° 59´ latitude. Soil samples were taken from the experiment field to measure its soil moisture content and soil chemical properties. These properties were measured by a Biochrom Libra S22 spectrophotometer and a Jenway Flame Spectrometer to indicate the sulfate and sodium of the soil. Soil pH and soil texture were measured by Hana 209 pH meter and a hydrometer. The chemical elements are determined using a burette titration along with a Jenway 4510 conductivity meter to monitor the electrical conductivity (EC) range of the soil. A penetrologger (Eijkelkamp, Netherland) with cone base area of 1 cm^2^ and a cone apex angle of 60° was used to measure vertical soil mechanical resistance (soil cone index (CI)). The sweep blades were attached on the tillage tool shanks. An ITMCO 399 tractor was used to pull the tillage tool in the field. The experiment conducted at a soil depth of 15 cm, with a tractor forward speed of 6 km/h at four traveling distances of 2, 4, 6 and 8 km. The first blade was taken off at 2 km traveling distance. Afterward, the second blade was taken off at 4 km, the third blade at 6 km and eventually, the final fourth blade at 8 km.

In order to perform a set of analysis, samples of 1 cm^2^ were cut off the blades. An Ara Research NANOVAC atomic force microscopy (AFM) and a Phenom-XL scanning electron microscopy (SEM) was utilized for surface morphology investigations. Normal X-ray diffraction (XRD) and both regional and multi-spot (Line Scan) energy-dispersive X-ray spectroscopy (EDX) (attached to the SEM device) were used for element detections and crystallographic studies. A Vickers Hardness tester was utilized to measure the hardness of the coated layers. The surface roughness measurements were carried out by Nano Pajouhan Raga Surface Profilometer 50.10.s and AFM. The blades draft force was measured in the soil bin^70^, at the depth of 15 cm and soil conditions similar to the field.

**Figure 7:**
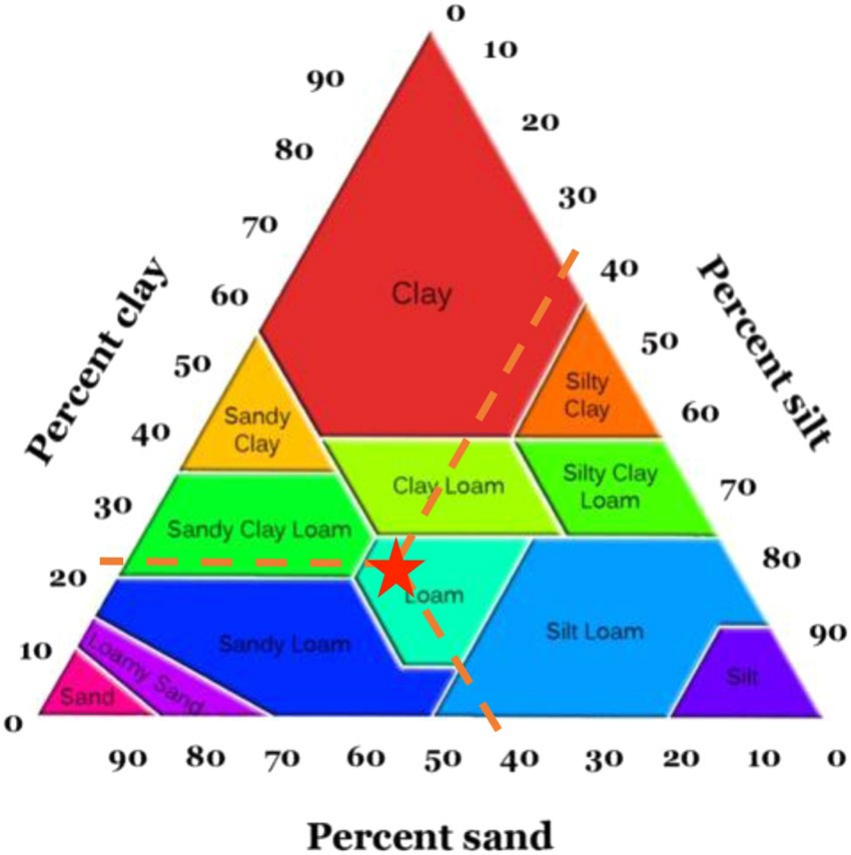
Soil texture triangle.

**Figure 8:**
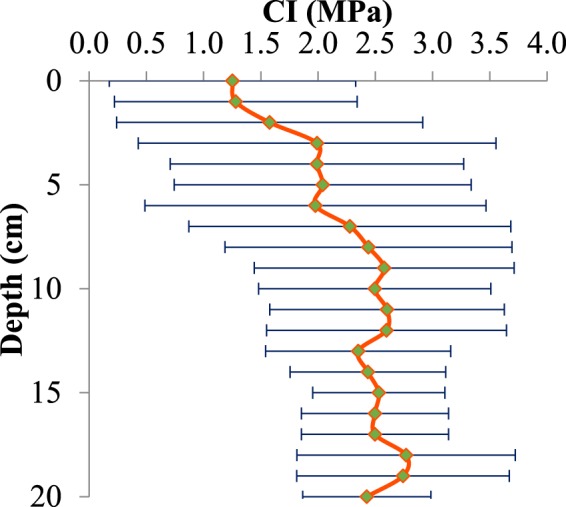
Soil mechanical resistance of the field (CI).

The experimental conditions same as previous study^1^. The soil physical and chemical properties of the experimental field were as given below. The number of total cations was 13.41 milliequivalent/liter (meq/lit) (Mg^2+^ = 4.5, Ca^2+^ = 5.75, Na^+^ = 3.17) and total anions was 15.41 meq/lit (HCO_3_^−^ = 6.25, Cl^−^ = 3.75, SO_4_^−2^ = 5.41). Soil electrical conductivity and soil pH were 1.36 ds/m and 7.62, respectively. Soil physical properties including soil texture, moisture content and soil mechanical resistance known as CI were also measured. The percentage of soil particles was 45% sand, 21% clay and 34% silt. The soil texture of the field was then determined as loam according to soil texture triangle^70,71^ (Fig. 7). Soil moisture content was 11%. Prior to carrying out the experiments in the field, soil CI was measured using penetrologger to a depth of 20 cm at 10 insertion points with the distance of 20 m from each other in the field to obtain the mean CI. The value of cone index increases with the depth of the soil. CI of the soil started from 1.25 MPa on the soil surface and reached to 2.5 MPa at the depth of 20 cm. The soil CI with the related variations is depicted in Fig. 8. The field had not been tilled for one year. Stones and crop residues were also observed in the field. A hard soil condition was chosen to test the blades at the harshest environment.

**Figure 9:**
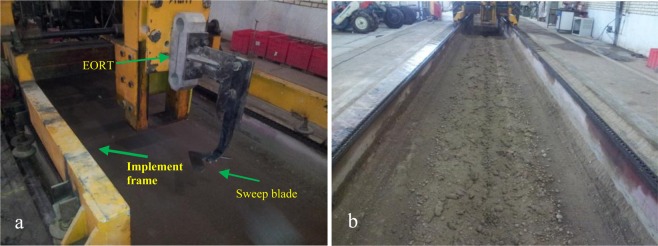
Extended octagonal ring transducer (EORT) (**a**) used for measuring draft force of sweep blade in the soil bin (**b**).

A soil bin equipped with an extended octagonal ring transducer (EORT)^72^, which is attached to an implement frame, is shown in Fig. 9. The EORT was used to measure the draft force of the sweep duck blades at 15 cm depth in the soil with the moisture content of 12%. The forward speed of the blades was 6 km/h. The optimum length of the soil bin was 9 m. The influence of the blades wear on the draft force was also investigated. Experimental design of factorial test based on the randomized complete design (RCD) was employed to perform the experiments. Two factors of traveled distance at five levels of 0, 2, 4, 6 and 8 km of both coated and uncoated blades with three replications were used for statistical analysis of draft force data. The mean draft force is compared with Duncan multiple range test at 95% level of confidence by SPSS statistical software (ver. 22).

## Conclusion

The multi-layers of TiCrN, TiAlN, TiAlSiN, and TiAlSiCN with the thickness of 4.2 μm have been deposited on CK45 carbon steel for performance improvement of tillage tools (duck blades). The arc-PVD method was able to apply high-quality layers to the samples with no need for post-annealing at high temperatures (XRD and EDX data supported this statement). The sweep blades were tested at both the lab (soil bin) and on the field to investigate their resistance to wear and draft force. With the comparison of the SEM cross-sectional image and the profilometer roughness the worn layers were calculated for most of the samples to be only about ~100 nm. The soil bin draft force experiment showed an increase of draft force as the coated layers wore off after 2 km distance. All in all, tillage tool blades resistance to wear, use full lifetime where increased due to the use of TiCrN-TiAlN-TiAlSiN-TiAlSiCN multi-layer coatings. This operation improvements are owned to the application of arc-PVD technique in deposition of hard multi-layer.
